# Effects of Nut Consumption on Blood Lipids and Lipoproteins: A Comprehensive Literature Update

**DOI:** 10.3390/nu15030596

**Published:** 2023-01-23

**Authors:** Marta Guasch-Ferré, Anne-Julie Tessier, Kristina S. Petersen, Philip A. Sapp, Linda C. Tapsell, Jordi Salas-Salvadó, Emilio Ros, Penny M. Kris-Etherton

**Affiliations:** 1 Department of Public Health, Novo Nordisk Foundation Center for Basic Metabolic Research, Faculty of Health and Medical Sciences, University of Copenhagen, 1014 Copenhagen, Denmark; 2 Department of Nutrition, Harvard T.H. Chan School of Public Health, Boston, MA 02115, USA; 3 Department of Nutritional Sciences, Texas Tech University, Lubbock, TX 79409, USA; 4 Department of Nutritional Sciences, Penn State University, State College, PA 16802, USA; 5 Faculty of Science, Medicine and Health, University of Wollongong, Wollongong, NSW 2522, Australia; 6 llawarra Health and Medical Research Institute, Wollongong, NSW 2522, Australia; 7 Unitat de Nutrició Humana, Departament de Bioquímica i Biotecnologia, Universitat Rovira i Virgili, 43204 Reus, Spain; 8 Institut d’Investigació Sanitària Pere Virgili (IISPV), 43204 Reus, Spain; 9 Consorcio Centro de Investigación Biomédica en Red CIBER, Fisiopatología de la Obesidad y Nutrición (CIBERObn), Instituto de Salud Carlos III (ISCIII), 28029 Madrid, Spain; 10 Lipid Clinic, Endocrinology and Nutrition Service and Institut d’Investigacions Biomèdiques August Pi Sunyer, Hospital Clínic, 08036 Barcelona, Spain

**Keywords:** nuts, cholesterol, lipids, apolipoproteins

## Abstract

In the present review, we provide a comprehensive narrative overview of the current knowledge on the effects of total and specific types of nut consumption (excluding nut oil) on blood lipids and lipoproteins. We identified a total of 19 systematic reviews and meta-analyses of randomized controlled trials (RCTs) that were available in PubMed from the inception date to November 2022. A consistent beneficial effect of most nuts, namely total nuts and tree nuts, including walnuts, almonds, cashews, peanuts, and pistachios, has been reported across meta-analyses in decreasing total cholesterol (mean difference, MD, −0.09 to −0.28 mmol/L), LDL-cholesterol (MD, −0.09 to −0.26 mmol/L), and triglycerides (MD, −0.05 to −0.17 mmol/L). However, no effects on HDL-cholesterol have been uncovered. Preliminary evidence indicates that adding nuts into the regular diet reduces blood levels of apolipoprotein B and improves HDL function. There is also evidence that nuts dose-dependently improve lipids and lipoproteins. Sex, age, or nut processing are not effect modifiers, while a lower BMI and higher baseline lipid concentrations enhance blood lipid/lipoprotein responses. While research is still emerging, the evidence thus far indicates that nut-enriched diets are associated with a reduced number of total LDL particles and small, dense LDL particles. In conclusion, evidence from clinical trials has shown that the consumption of total and specific nuts improves blood lipid profiles by multiple mechanisms. Future directions in this field should include more lipoprotein particle, apolipoprotein B, and HDL function studies.

## 1. Introduction

Cardiovascular diseases (CVD), specifically coronary heart disease (CHD) and stroke, are leading causes of death and disability-adjusted life years worldwide [1]. Dyslipidemia (elevated levels of low-density lipoprotein cholesterol (LDL-cholesterol) or triglycerides (TG) in blood, decreased levels of high-density lipoprotein cholesterol HDL-cholesterol, or other lipoprotein disturbances) is a well-documented risk factor for the development of atherosclerotic CVD [2]. CVD and its related risk factors are largely preventable. Therefore, effective approaches for the prevention of CVD, including changes in lifestyle and diet, are key to reducing the consequences of dyslipidemia and the associated disease burden to improve population health.

Dietary interventions to lower blood cholesterol concentrations and modify blood lipoprotein levels are the cornerstone of prevention and treatment for CHD and other atherosclerotic diseases. Indeed, suboptimal dietary intake was responsible for an estimated one in five premature deaths globally from 1990 to 2016 [3]. In the United States (U.S.), suboptimal diets were associated with more deaths than any other risk factor. In 2016, dietary risk factors were responsible for an estimated 529,300 deaths, of which 84% were due to CVD [4]. Among individual dietary components, the largest estimated mortality was associated with an excessive sodium intake (9.5%) followed by the suboptimal consumption of nuts/seeds, among others [5]. In addition, in 2017 a diet low in nuts and seeds was the fourth leading risk factor for all-cause mortality globally, after a diet low in whole grains, a diet high in sodium, and a diet low in fruits [5].

Nuts and seeds, along with other plant foods such as whole grains, vegetables, fruits, and legumes, are key components of recommended healthy diet patterns worldwide such as the Mediterranean diet. Nuts are a good source of unsaturated fatty acids and are rich in fiber, minerals (potassium, calcium, and magnesium), vitamins (folate and vitamin E), phytosterols, and polyphenols. The fatty acid composition varies widely among different types of nuts [6]. Almonds, hazelnuts, pistachios, cashews, and peanuts are rich in monounsaturated fatty acids (MUFAs), whereas walnuts are rich in polyunsaturated fatty acids (PUFAs) [6,7].

In recent decades, an extensive body of evidence has linked nut consumption to a wide range of health benefits including reduced risk and prevention of cardiometabolic diseases [8], making them a key dietary recommendation for health promotion and disease reduction. In large prospective cohort studies, frequent nut consumption has been inversely associated with the risk of type 2 diabetes, metabolic syndrome, CVD, and total and cause-specific mortality [8]. These findings are consistent with the results of the PREvención con DIEta MEDiterránea (PREDIMED) study, a primary prevention trial that found a 28% reduction in incident cardiovascular events among participants randomly assigned to a Mediterranean diet supplemented with nuts [9]. In addition, short-term trials have demonstrated the beneficial effects of nut consumption on intermediate markers of CVD risk, including LDL-C [10]. Importantly, more than 60 human dietary intervention studies have been conducted investigating the effects of nut consumption on blood lipid levels. These studies differ in the type and quantity of the nuts consumed, placebo/diet control, study design, subject selection criteria, and duration.

In the present narrative review, we provide a comprehensive overview of the current knowledge on the effects of total and specific types of nut consumption (excluding nut oil) on blood lipids and lipoproteins in clinical trials, the potential mechanisms of the lipid effects of nuts, and the future directions for research in this area. 

## 2. Effects of Nuts on Blood Lipids

We conducted a literature search in PubMed (https://pubmed.ncbi.nlm.nih.gov/, accessed on 22 December 2022) for systematic reviews and meta-analyses of randomized controlled trials (RCTs) that examined the effect of nut consumption on blood lipid biomarkers from the inception date through 20 November 2022. The search strategy was as follows: (nuts[MeSH] OR “tree nuts” OR nut OR almonds[MeSH] OR almond OR walnuts[MeSH] OR walnut OR cashews[MeSH] OR cashew OR pistachios[MeSH] OR pistachio OR peanuts[MeSH] OR peanut OR “peanut butter”) AND (meta-analysis OR “systematic review”) AND (English[lang]). We restricted the search to adult human trials and to articles published in English. Selected articles were required to focus on whole nuts or nuts-enriched food interventions, and to report on at least one lipid variable (TC, LDL cholesterol, HDL cholesterol, TGs, or apolipoproteins). We excluded articles that included nut oils as an intervention given their different nutrient matrix.

Numerous systematic reviews and meta-analyses of RCTs that evaluated nut consumption on blood lipid biomarkers have been published [11,12,13,14,15,16,17,18,19,20,21,22,23,24,25,26,27,28,29]. Table 1 summarizes 19 selected studies. Three focused on interventions of both nuts and peanuts together [11,12,21], three were on tree nuts [16,18,22], five on almonds [13,15,19,25,29], four on walnuts [17,23,24,26], two on cashews [20,28], one on pistachios [27] and one on peanuts including peanut butter [14]. The doses tested varied from 5 to 200 g/d and the study durations were from 4 days to 2 years. 

A significant reduction in LDL-C (mean differences, MD: −0.09 to −0.26 mmol/L; 10 of the 18 meta-analyses analyzing LDL-C) was most consistently reported across meta-analyses, followed by a reduction in triglycerides (TG; MD: −0.05 to −0.17 mmol/L; 9/19 meta-analyses) and total cholesterol (TC; MD: −0.09 to −0.28 mmol/L; 8/19). However, none reported an effect on HDL-C. Del Gobbo et al. (2018) further examined the effect of nuts, specifically tree nuts, on apolipoproteins (Apo) and found a significant reduction in Apolipoprotein B (ApoB) (−0.042 g/L (95% CI: −0.057, −0.026); 13 RCTs) [22]. In one meta-analysis that reported significance, the observed effect of nuts on LDL-C was comparable to up to ¼ of the effect of statin medication in populations including primary prevention, hemodialysis, CHD, diabetes, heart failure, and in those at low vascular risk [30,31].

Although the evidence supports a modest effect of nuts in lowering blood lipids/lipoproteins, it is unclear whether some types of nuts are more effective than others. Among tree nuts, walnuts are especially rich in linoleic acid (18:2n–6) and α-linolenic acid (18:3n–3) (ALA) [6]. The meta-analyses demonstrated the beneficial effects of walnuts on reducing TC, LDL-C, and TG [17,18,23,24]. This finding corroborates previous studies showing slightly reduced fasting serum TG [MD: −0.03 mmol/L (−0.11, −0.05)] with increasing ALA intake [32]. Pistachios are particularly rich in phytosterols and dietary fiber, and are high in MUFAs [33,34]. The only meta-analysis (of 12 RCTs) on pistachios found a significant effect of 32–126 g/d during 3–24 weeks in reducing TC (−0.19 mmol/L [95% CI: −0.33, −0.06]), LDL-C (−0.1 mmol/L [95% CI: −0.14, −0.06]), and TG (−0.13 mmol/L [95% CI: −0.16, −0.09]) [27]. Like pistachios, cashews have a high proportion of MUFAs but are lower in tocopherols, phytosterols, and dietary fiber. Few studies have examined the effect of cashew consumption on blood lipids. Two meta-analyses did not find any effect of 28–108 g cashews/d on lipid biomarkers in adult populations (3 RCTs; n = 384 to 392, duration: 4–12 weeks) [20,28]. These results were also confirmed in another meta-analysis [18]. The absence of an effect may be attributed to its differing food matrix or to limited available studies. Almonds are especially rich in alpha-tocopherol [34] and dietary fiber compared with other nuts. A consistent beneficial effect of almonds (10–168 g/d; 5–27 RCTs, n = 120–2,049 healthy or at risk of CVD individuals; 3–77 weeks duration) was reported in LDL-C (−0.15 to −0.18 mmol/L) [13,19,25], TC (−0.13 to −0.28 mmol/L) [19,25], and TG (−0.08 mmol/L) [25]. However, the evidence is less consistent in populations with type 2 diabetes [15,29]. Peanuts, although classified as a legume, have a comparable food matrix and fatty acid composition to those of tree nuts. The effect of 25–200 g/d peanuts or peanut butter consumption during 2–24 weeks on blood lipids was examined in a recent meta-analysis and demonstrated a significant reduction in TG [−0.13 mmol/L (95% CI: −0.2, −0.07)]; 9 RCTs, and 643 participants) [14].

To our knowledge, one RCT was published following the last meta-analysis on nuts and blood lipids that we summarize herein. The Brazilian Nut Study tested the effect of an energy-restricted diet with 45 g nuts (15 g Brazil nuts + 30 g cashews) and without nuts on various biomarkers including blood lipids in 40 women at risk of cardiometabolic disease [35]. After the 8-week intervention, the authors reported a decrease in TC and LDL-C in both groups, but no difference between groups; this is possibly due to the significant weight loss achieved in both the intervention (−3.5 +/−0.6 kg; *p* < 0.001) and the control (−1.8 ± 0.6; *p* < 0.05) groups at the end of the trial.

### 2.1. Dose-Response Effects of Nuts on Blood Lipids

In eight of the 19 identified meta-analyses, dose-response analyses were conducted [12,16,22,23,24,25]. Some evidence suggests nuts dose-dependently improve TC, LDL-C, and TG. In a meta-analysis of 61 clinical trials, Del Gobbo et al. reported that tree nut intake lowered TC and LDL-C in a nonlinear manner such that stronger effects were observed in trials where >60 g/d of tree nuts were provided. For ApoB and TGs, linear dose-response effects were observed [22]. Similarly, Blanco Mejia et al. observed a borderline non-significant linear relationship between increasing tree nut doses and TG reductions; no dose-response relationship was observed for HDL-C [16]. In a pooled analysis of 25 RCTs, Sabaté et al. observed dose-dependent reductions in TC, LDL-C, and TG with higher consumption of tree nuts and peanuts [12].

In five meta-analyses, dose-response analyses were conducted to examine the relationship between the intake of a single nut type and lipid/lipoprotein responses [14,23,24,25,26]. In a meta-analysis of 24 clinical trials, higher walnut intake was dose-dependently associated with reductions in TC (−0.01 mmol/L per 1 g/d increase) [23]. A trend towards a dose-response relationship was observed for LDL-C (−0.01 mmol/L per 1 g/d increase), while no dose-response relationship was observed for TG or HDL-C. A recent meta-analysis of five studies conducted in cohorts with metabolic syndrome showed a non-linear association between walnut consumption and HDL-C whereby an intake of up to 50 g/d was associated with an increase in HDL-C [26]. Additionally, a trend toward a linear dose-response relationship was observed between the consumption of walnuts and TG reduction; no dose-response relationship was observed for TC or LDL-C. Less dose-response specific evidence is available for other nuts including almonds and peanuts. In a meta-analysis examining the effect of almond intake on lipids and lipoproteins, an inverse linear relationship was observed between almond dose and TG. TC, LDL-C, and HDL-C were not linearly related to almond intake dose [25]. A meta-analysis of nine RCTs showed no dose-response relationship between peanut intake and TC, LDL-C, HDL-C, or TG [14].

In 11 of the 19 included meta-analyses, subgroup analyses were conducted to examine the effect of higher vs. lower consumption of nuts on lipid/lipoprotein levels [11,13,15,16,17,19,21,23,25,26,29]. The highest consumption category was >63 g/d in one meta-analysis [26], ≥50 g/d in five meta-analyses [13,15,16,21,29], ≥45g/d in two meta-analyses [11,25], >42.5 g/d in one meta-analysis [19], ≥42 g/d in one meta-analysis [17], and ≥28 g/d in one meta-analysis [23]. In a meta-analysis of studies on healthy individuals, the consumption of ≥45 g/d of almonds lowered TC, LDL-C, and TG to a greater extent than <45 g/d [25]. Similarly, in a meta-analysis of five studies conducted in patients with type 2 diabetes, greater reductions in TC, LDL-C, HDL-C, and TG were observed with the consumption of ≥50 g/d of almonds [29]. In two meta-analyses including healthy participants and individuals at high risk for CVD, no effect modification by almond dose (≥50 g/d and >42 g/d) was observed [13,19].

Three meta-analyses examined the effect of walnut dose levels on changes in lipids and lipoproteins [17,23,26]. Guasch-Ferré et al. reported similar TC and LDL-C lowering with a walnut consumption of ≥28 g/d compared with <28 g/d [23]. However, greater reductions in TC and LDL-C were observed when walnut intake comprised 10–25% of total energy compared with 5–10% of total energy. No dose-related effect modification was observed for TG or HDL-C. In a meta-analysis of studies including middle-age and older adults, TC and TG reductions were only observed when walnut consumption was ≥42 g/d; LDL-C was lowered to a similar magnitude at both doses [17]. In a meta-analysis of studies including participants with metabolic syndrome, walnut dose (>63 g/d vs. ≤63 g/d) did not affect LDL-C differently [26].

Inconsistent findings were reported in three meta-analyses examining lipid/lipoprotein effect modification by doses of tree nuts and peanuts or tree nuts only [11,16,21]. A meta-analysis including studies involving patients with type 2 diabetes showed that higher tree nut and peanut consumption (≥45 g/d) lowered TC and LDL-C significantly, whereas lower consumption was not associated with TC and LDL-C lowering [11]. In contrast, in a meta-analysis of 11 studies including participants with overweight/obesity, no difference in lipid responses by peanut and tree nut dose (≥50 g/d vs. <50g/d) was observed [21]. In a meta-analysis of studies conducted in participants that were healthy or had dyslipidemia, metabolic syndrome, or type 2 diabetes, TGs were reduced with a higher intake of tree nuts (≥50g/d); HDL-C findings were not different by the dose consumed and LDL-C and TC were not assessed [16].

Collectively, there is evidence supporting that nuts dose-dependently improve lipids and lipoproteins. However, many of the meta-analyses reviewed included a relatively small number of studies, which limits the statistical power to examine dose-response relationships. In addition, across the meta-analyses reviewed, higher vs. lower consumption was inconsistently defined, and limited rationale was provided in most cases for the cut points used.

### 2.2. Subgroup Analyses: Effects of Nuts on Blood Lipids

Across the meta-analyses reviewed, several subgroup analyses were conducted to assess the potential for sex, age, BMI, baseline lipid/lipoprotein concentrations, and health status to influence the effect of nuts on lipids and lipoproteins. Broadly, sex and age do not appear to be effect modifiers; BMI and baseline lipid/lipoprotein concentrations may influence the lipids/lipoprotein lowering effects of nuts.

#### 2.2.1. Sex

In two of the included meta-analyses, subgroup analyses evaluating effect modification by sex were reported [12,22]. In both meta-analyses, no differences in the effect of nuts on lipids/lipoproteins were observed by sex [12] or by the proportion of the study sample that was men (≥50% or <50%) [22].

#### 2.2.2. Age

Seven of the included meta-analyses conducted subgroup analyses to assess the effect of nuts on lipids/lipoproteins in different age categories [11,12,17,22,23,25,26]. Limited evidence suggests that TG lowering in response to tree nut intake may be greater in those aged <50 years; however, most of the evidence evaluated suggests age is not a strong effect modifier. In four meta-analyses, including studies examining the effect of tree nuts and peanuts [12], tree nuts only [22], and walnuts [23,26] on lipids and lipoproteins, effects did not differ across age categories. In two meta-analyses, TG reductions were only observed in response to almond [25] and walnut [17] consumption in the <50 y age category; no intervention effect was observed in ≥50 y age category. In both meta-analyses, no differences in TC or LDL-C were observed by age category. Xia et al., however, observed only TC lowering in response to tree nut and peanut consumption in the ≥55 y age category; there was no difference in LDL-C by age category [11].

#### 2.2.3. BMI

Across the five meta-analyses that conducted subgroup analyses to evaluate effect modification by BMI, the evidence suggests nuts may induce greater lipid/lipoprotein improvements when BMI is <30 kg/m^2^ [12,15,21,23,25]. Sabaté et al. observed greater improvements in the LDL-C/HDL-C ratio and TC/HDL-C ratio when BMI was <25 kg/m^2^ and 25–30 kg/m^2^ compared with BMI >30 kg/m^2^ [12]. Similar trends were observed for LDL-C, TC, and TG. Eslami et al. reported TG lowering with the consumption of tree nuts and peanuts only when BMI was <30 kg/m^2^; in this meta-analysis effect modification was not observed for TC, LDL-C, and HDL-C [21]. Guasch-Ferré et al. did not observe any difference in the effect of walnuts on lipids/lipoproteins in studies where BMI was <25 kg/m^2^ vs. ≥25 kg/m^2^ [23]. Similarly, Moosvian et al. reported that the effect of almonds on lipids and lipoproteins in studies including patients with type 2 diabetes did not differ by BMI category (<30 vs. ≥30 kg/m^2^) [15]. Conversely, in a meta-analysis including studies conducted in generally healthy populations, reductions in TC and LDL-C were only observed with almond consumption in individuals with overweight; no differences in TG or HDL-C were observed across BMI categories [25].

#### 2.2.4. Baseline Lipids/Lipoprotein Concentrations

In nine of the included meta-analyses subgroup analyses were conducted to examine effect modification by baseline lipid/lipoprotein concentrations or hyperlipidemia/dyslipidemia status [12,13,15,16,22,23,24,25,27]. Evidence suggests greater improvements in lipids and lipoproteins in response to nut consumption when the baseline TC and/or LDL-C is higher [12,25,27] or in participants with hyperlipidemia or dyslipidemia [13]. However, in meta-analyses that examined effect modification by baseline TG concentrations, reductions in TGs were only observed when the baseline TG concentrations were lower (<1.69 mmol/L) [16,25,27]. In four meta-analyses, no effect modification was observed by the baseline lipid/lipoprotein level or the hyperlipidemia/dyslipidemia status [15,22,23,24]. Across the evaluated meta-analyses, inconsistent cut points were used to define higher vs. lower baseline lipid/lipoprotein concentrations and dyslipidemia/hyperlipidemia, which likely contributes to the variability observed.

#### 2.2.5. Health Status

Across the six meta-analyses that conducted subgroup analyses to assess effect modification by health status (healthy vs. metabolic impairment), inconsistent findings were reported, with no clear pattern of effect modification by health status [14,17,19,22,25,27]. In the largest meta-analysis including 61 trials, no heterogeneity in the effect of tree nuts on TC, LDL-C, HDL-C, or TG was observed by disease status (healthy, type 2 diabetes, metabolic syndrome, high cholesterol, obesity) [22]. In this analysis, ApoB reductions were greater in those with type 2 diabetes (−0.115 g/L, 95% CI −0.162, −0.068) compared with healthy populations (−0.025 g/L; 95% CI −0.047, −0.003). Given the variation in the methodology used in these meta-analyses and the aggregate nature, subgroup analyses have limited power to identify true differences between subgroups. To further explore effect modification by health status, individual participant data meta-analyses are needed.

### 2.3. Effects of Nut Processing on Blood Lipid Profile

Few investigations of the effect of nut processing on lipids/lipoproteins have been conducted. The available evidence from RCTs suggests almond [36,37], hazelnut [38], and peanut [14,39,40] processing (i.e., roasting, or production of oil or butter) does not alter lipid and lipoprotein responses. In an RCT including adults with normolipidemia, the consumption of ~14% of energy from almond oil or whole almonds for 6 weeks improved TC, LDL-C, HDL-C, and TG with no difference in almond processing [36]. Similarly, in a study of individuals with hypercholesterolemia, the consumption of 100 g/d of roasted salted almonds, roasted almond butter, or raw almonds improved TC and LDL-C after 4 weeks and the magnitude of the effect was not impacted by almond processing [37]. Comparable findings were observed in an RCT whereby the intake of 30 g/d of either raw or dry roasted, lightly salted hazelnuts for 4 weeks did not differentially affect TC and LDL-C [38].

Findings from three RCTs suggest peanut processing does not influence lipid/lipoprotein changes [14,39,40]. In a five-arm randomized trial, the intake of 56 g of whole raw unsalted peanuts, whole roasted unsalted peanuts, whole roasted salted peanuts, whole honey roasted peanuts, or peanut butter for 4 weeks did not affect TC, LDL-C, HDL-C, or TG differently [40]. This is consistent with findings from crossover RCTs where the intake of a diet enriched with peanut butter/peanuts similarly improved TC, LDL-C, TG, and ApoB compared with an average American diet [39]. Similar findings were observed in a 6-month RCT examining the intake of 25 g/d of skin-roasted peanuts, two tablespoons (32 g)/day of peanut butter, or two tablespoons (32 g)/day of peanut oil [14]. In this trial, no differences in TC, LDL-C, HDL-C, or TG were observed per peanut form. Thus, from the limited evidence available, nut processing does not appear to alter lipid/lipoprotein responses.

## 3. Proposed Mechanisms of Action of Cholesterol-Lowering by Nuts

**Figure 1:**
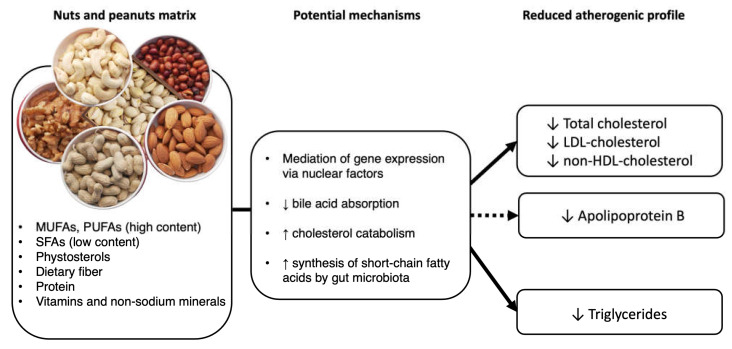
Potential mechanisms by which tree nuts and peanuts reduce atherogenic lipid/lipoprotein profile. A plain arrow indicates a strong level of evidence to support the effect, whereas a dashed arrow indicates a lower level of evidence. LDL, low density lipoprotein; HDL, high density lipoprotein.

Nuts are a good source of MUFAs and PUFAs, and they also contain dietary fiber, phytosterols, and polyphenols. In isolation, all these nutrients and bioactive compounds may have a modest cholesterol-lowering effect; however, when these molecules combine in the matrix of nuts and synergize to potentiate cardiometabolic pathways, they have the capacity to reduce LDL-cholesterol beyond the effects predicted by equations based solely on fatty acid profiles [41]. Figure 1 summarizes the potential mechanisms for the beneficial effects of nuts consumption on lipid metabolism with the ensuing reduction of the atherogenic lipid/lipoprotein profile.

Specifically, nuts have favorable effects on serum lipids primarily because of their high content of unsaturated fatty acids (both MUFAs and PUFAs), while they have a low content of saturated fatty acids (SFAs; 4 to 15%) [42]. The unique fatty acid profile of nuts facilitates a favorable shift in the dietary fatty acids when nuts are substituted for foods that are high in SFAs or carbohydrates. Dietary PUFAs have been shown to reduce ApoB while MUFAs increase ApoA1, which mediates the efflux of cholesterol associated with HDL particles [41]. Experimental and clinical studies have shown that the intake of unsaturated fatty acids enhances the hepatic receptor-dependent clearance of LDL and concomitantly reduces plasma LDL-C levels [43]. Unsaturated fats from nuts replacing SFA in lipid bilayers increase membrane fluidity, flexibility, and elasticity, while reducing membrane thickness [44]; these physical changes impact the interaction of membrane-bound receptors with their ligands, such as the affinity of LDL receptors for ApoB-100 in LDL particles, thus enhancing LDL-C uptake. Additionally, PUFA can mediate the expression of several genes involved in lipid metabolism via nuclear factors, including the peroxisomal proliferator-activated nuclear receptors gamma (PPARγ), liver X-receptor (LXR), hepatocyte nuclear factor-(HNF)-4α, nuclear factor kappa B (NFκB), and sterol-regulatory element binding proteins (SREBPs) [45]. Particularly, PUFAs downregulate the expression of SREBPs and enzymes for cholesterol synthesis, thus decreasing the body cholesterol pool [45].

Improvement in blood lipids is attributable mainly to the favorable fatty acid profile of nuts, but other nut components, namely dietary fiber, and plant sterols [42], may also play a significant role. Nuts contain ~7 g/100 g dietary fiber, of which ~25% is soluble fiber [46]. In a meta-analysis of 67 clinical trials to quantify the cholesterol-lowering effect of dietary fiber, 2–10 g/d of soluble fiber was associated with modest but significant reductions in total cholesterol and LDL-C [47]. According to a recent umbrella meta-analysis, total dietary fiber (independently of type) also has cholesterol-lowering properties [48]. Dietary fiber, particularly soluble fiber, exerts its hypocholesterolemic effect through several mechanisms: (1) increased intestinal viscosity, which reduces bile acid absorption and promotes cholesterol catabolism; (2) enhanced synthesis of short-chain fatty acids by gut microbiota, particularly butyrate and propionate, which reduce de novo cholesterol synthesis via 3-hydroxy-3-methylglutaryl-CoA (HMG-CoA) reductase inhibition; and (3) interference with micelle formation in the intestinal lumen and enhanced fecal excretion of fat, cholesterol, and bile acids [48].

Like all plant foods, nuts are cholesterol-free, but their fat fraction contains chemically related plant sterols or phytosterols [42]. The phytosterol content is variable ranging from approximately 72 to 272 mg/100 g with pistachios, almonds, and walnuts containing the most. These compounds play a structural role in their cell membranes just as cholesterol does in animal cell membranes [49]. Phytosterols interfere with cholesterol absorption in the intestinal lumen and thus help lower blood cholesterol. Interestingly, evidence has demonstrated that phytosterols contribute to the cholesterol-lowering effect of nut consumption [50]. In a systematic review and meta-analysis of 61 trials with 2582 participants, with nut intakes from 0.2 to 3.5 servings/d (equivalent to about 5 to 100 g/d) and phytosterol doses ranging from 4.8 to 279 mg/d, the total phytosterol dose from nuts was inversely correlated with a decrease in LDL-C (r = –0.60) [50]. Of note was that the predominant LDL-C lowering effect was due to the quantity of nuts consumed; hence representing a greater quantity of both phytosterols and unsaturated fat consumed. A review by Cofan and Ros [51] summarized the LDL-C lowering effects of phytosterols and reported that one meta-analysis [52] concluded that the cholesterol-lowering effects were greater when phytosterols were consumed with fat in the food matrix. Collectively, the evidence suggests that the LDL-C lowering effects of nuts are primarily due to their fatty acid profile but also due to the effects of dietary fiber and plant sterols, as well.

Finally, beyond LDL-C lowering, nuts contain highly bioactive polyphenols, representing one of the richest food sources [42,53]. Although data from clinical studies are few and inconclusive, the lipid effects of polyphenols appear to be limited to reducing LDL oxidation [54], which concurs with the well-known antioxidant and anti-inflammatory properties of these phytochemicals and likely contributes to their atheroprotective role. Oxidized LDL (oxLDL) is important because it is involved in several steps of atherogenesis (endothelial injury, leukocyte recruitment/retention, foam cell formation, etc.) [55,56]. However, the evidence for the benefits of nut consumption on oxLDL and other biomarkers of LDL oxidation is inconsistent, as shown in a recent review of 12 RCTs using a variety of nuts and showing results for changes in blood oxidation markers, usually as secondary outcomes [56].

Another important consideration of the mechanisms to explain the health benefits of nuts pertains to their effects on reverse cholesterol transport, an important mechanism whereby cholesterol is removed and transported from peripheral tissues by HDL to the liver for disposal. A recent review on the role of HDL in atherosclerotic disease notes the shift from measuring HDL-C concentrations to focusing on the more functional measures of HDL (e.g., HDL particle number and cholesterol efflux capacity), which is more predictive of future atherosclerotic CVD events [57]. There is evidence that nuts increase HDL function (i.e., increased cholesterol efflux capacity). This has been demonstrated for walnuts, pistachios, and mixed nuts (walnuts, almonds, and hazelnuts) [58,59,60]. Nut consumption may shift HDL distribution to improve reverse cholesterol transport. A shift, or increase, in large HDL particles, could indicate increased reverse cholesterol transport due to their affinity to sequester cholesterol that effluxes from macrophages via ATP Binding Cassette Subfamily G Member 1 (ABCG1) [61]. Although the evidence is limited and the underlying mechanisms are not completely understood, nut intake may increase the reverse cholesterol transport capacity of HDL leading to increased removal of cholesterol from peripheral tissues.

Lipoprotein(a) [Lp(a)] is an independent, causal, risk factor for atherosclerotic CVD [62]. Lp(a) concentrations are primarily genetically predetermined with minimal effects from dietary interventions [62]. Several RCTs have assessed the effects of diets enriched with nuts at doses of 1.5 servings (42.5 g)/d or more compared with control diets in various populations (healthy, type 2 diabetes, hyperlipidemia, at risk of CVD). The results have been inconsistent, with modest reductions of Lp(a) in three trials using walnuts [63], pecans [64], or almonds [65], and no discernible effect in four further trials, two with almonds [66,67] and two with walnuts [68,69].

### 3.1. Emerging Evidence of the Effects of Nuts on Lipoprotein Particle Size

Lipoproteins, assessed by nuclear magnetic resonance, are categorized by particle sizes and densities, or lipoprotein subclasses, and many of these subclasses are associated with CVD outcomes [63]. A review by Qiao et al. on the role of LDL-C and LDL particles in atherogenesis concluded that LDL particles/density (e.g., oxLDL and small dense LDL) may be superior to LDL-C for predicting atherosclerotic CVD risk [64]. In fact, all ApoB-containing lipoproteins are atherogenic, and the small dense LDL particles are even more proatherogenic than larger LDL particles. In the Women’s Health Initiative, large very low-density lipoprotein (VLDL) particles increased CVD risk more so than small VLDL particles [65]. Interestingly, lipoprotein particle sizes are being evaluated in diet and nut studies. Observational studies and clinical trials have demonstrated a consistent relationship between improved diet quality and less atherogenic lipoprotein subclass profiles (lower large VLDL, small HDL, and small dense LDL) [66,67,68]. Moreover, the evidence to date indicates beneficial effects of nut consumption on lipoprotein profiles including particle sizes.

A cross-sectional and longitudinal analysis was conducted with 196 participants in the PREDIMED-Reus center to evaluate the associations of dietary intake (assessed by food frequency questionnaire [FFQ]) and plasma lipoprotein profiles at baseline and 1 year of follow-up [69]. Nut consumption for tertile 3 (highest nut consumption) was 26 g/d (total nuts); 14 g/d (walnuts); and 15 g/d (non-walnut nuts). The authors reported that the increased consumption of total nuts, walnuts, and non-walnut nuts was associated with decreased total and medium LDL particles, very large VLDL, and LDL-C; and decreased VLDL particle size, as well as increased HDL particles and HDL-C.

The Walnuts and Healthy Aging Study (WAHA), a multicenter study conducted in Spain and the U.S. with 628 participants (average age = 69 years) evaluated the effects of walnut consumption (15% of energy and 30 to 60 g/d) for two years on lipid and lipoproteins, including lipoprotein particle sizes [70]. The authors reported that the walnut diet decreased total LDL particles and small LDL particle numbers by 4.3% and 6.1%, respectively. In addition, the walnut diet significantly decreased total cholesterol, LDL-C, and intermediate-density lipoprotein cholesterol by 4.4%, 3.6%, and 16.8%, respectively. The take-home message from the WAHA Study is that the decrease in total LDL particles and small LDL particle number provides mechanistic insight into their cardiovascular benefit beyond changes in the conventional lipid/lipoprotein profile.

Several smaller clinical studies have shown similar benefits of tree nuts on lipoprotein particle size; however, likely because of the smaller sample sizes (and lower statistical power), significant diet effects were not consistently observed [59,71,72]. A study conducted with almonds (42.5 g/d) and dark chocolate (18 g cocoa power; 43 g dark chocolate/d) for four weeks reported that the almond diet significantly decreased LDL_1+2_, and the dark chocolate plus almond diet significantly decreased LDL_3+4_ compared with the average American diet [71]. In another study conducted by Tindall et al. [72], a diet that provided 18% energy from walnuts (57–99 g/d) tended (*p* < 0.1) to decrease LDL subclasses LDL_1+2_ and LDL_4_ compared with a fatty acid-matched diet and a diet where oleic acid was substituted for ALA in the comparator diets. Moreover, in a study conducted with pistachios [59] there was a significant decrease in small and dense LDL particles in response to a diet that provided 20% energy from pistachios (63–126 g/day) versus a diet with 10% of energy from pistachios as well as a lower-fat (25% of energy), low saturated fat (<8% of energy) control diet after 4 weeks. In addition, based on analysis of variance, there was a trend for an increase in ⍶-1 and ⍶-2 HDL (i.e., larger HDL particles) with the inclusion of pistachios. However, in a study conducted by Hernández-Alonso et al. [73] in participants with pre-diabetes, pistachios (57 g/d) increased small HDL particles and decreased medium and large HDL particles. The differences reported between the studies conducted by Holligan et al. [59] and Hernández-Alonso et al. [73] may be explained by differences in the study populations (i.e., healthy vs. pre-diabetes) and the known effects of elevated glucose levels on HDL function and HDL-C levels [74].

While research is still emerging about the effects of nuts on lipoprotein subparticle distribution and concentration, it is becoming clear that nuts favorably affect the conventional lipoprotein profile (i.e., reduced atherogenicity) with a consequent decreased risk of CVD. These findings are expanding our understanding of how tree nuts modulate lipoprotein metabolism and lower CVD risk.

### 3.2. Effects of Nut Consumption on Adiposity

Nuts are energy-dense foods containing high amounts of fat, a reason why there has been concern that their consumption may lead to weight gain and obesity. However, there is consistent evidence from large prospective studies, scientifically sound RCTs, and meta-analyses thereof that incorporation of substantial amounts of nuts into healthy diets do not lead to weight gain or increase the risk of abdominal obesity, and may even help promote weight loss and reduce waist circumference [75,76,77,78]. Several mechanisms explain the lack of the fattening effect of nuts, ranging from the effort required at mastication and chewing to increased satiety and the promotion of fullness due to delayed gastric emptying by the high fat and fiber content. Furthermore, the efficiency of energy absorption from nuts is reduced due to incomplete mastication and fat encasement within the unbroken cell walls in nut particles, thus limiting the bioaccessibility of fat from nuts in the intestine, with an ensuing increase in fecal fat losses [79].

## 4. Future Directions for Research on Nut Consumption and Blood Lipids

Non-communicable diseases such as CVD have multiple interacting dietary determinants, thus the effects of diet are likely to be dependent on the combination of foods rather than a single food [80]. Nevertheless, dyslipidemia remains a major risk factor contributing to CVD and the evidence supporting the effects of nut consumption on blood lipids and lipoproteins is compelling. Nut consumption improves lipid profiles by multiple mechanisms, and this understanding lays the groundwork for further research.

One of the challenges for this research is to integrate understanding at the level of key nutrients, foods, and dietary patterns. For example, a recent prospective study in the Coronary Artery Risk Development in Young Adults (CARDIA) study showed that individuals with higher walnut consumption also had higher diet quality (measured with Healthy Eating Index 2015), but also lower body mass index, waist circumference, blood pressure, and triglyceride concentration, and gained less weight since baseline than other nut consumers [81]. From a nutrient perspective, nuts make important contributions of unsaturated fatty acids, tocopherols, phytosterols, and dietary fiber. The relative composition varies by nut type, and this may explain inconsistencies in the research results. Separate studies may be required for mechanistic studies at the nutrient level, for example, on the role of PUFAs from nuts on gene expression related to lipid metabolism [44]. Further exploration of the effect of nut consumption on lipid particle number and size may need to focus on differences in fatty acid profiles and the varying doses of phytosterols and fiber provided by different nuts. This may be the case as the research progresses from the study of basic lipid profiles to sub-fractions, HDL function studies, and investigations around changes in ApoB.

Variations in results remain a problem for meta-analyses but this is often due to differences in study design, including dietary methodology. While age and sex do not appear to influence the effects of nut consumption on lipids, weight changes can confound results, so total diets are important. Likewise, studies may show that the processing of nuts does not appear to influence their relationship to blood lipids, but it may influence weight, due to the increased available energy from processing [82], so food form remains a consideration.

Other study design issues relate to the need for greater power in studies (larger sample sizes), further investigation of the linearity of effects (and determination of cut points), and study populations’ health status. Given the multifaceted effects on blood lipids and the variations in disease profiles of study participants, more individual participant data meta-analyses may be required.

Further studies evaluating the association between nut consumption and the microbiome are needed [83,84], a new horizon for research with the potential to add to our knowledge of how nuts influence lipid profiles. Preliminary reports indicate little change, but modulatory effects are emerging. On the other hand, plasma metabolomics are providing a useful innovative path for research linking nut consumption with CVD risk [85] and providing insights into the underlying mechanisms. This provides added support for the growing evidence of the effects of nut consumption on lipids and lipid fractions.

## 5. Conclusions

In conclusion, evidence from clinical trials has shown that the consumption of total nuts and specific types of nuts improves blood lipid profiles by multiple mechanisms, as discussed herein. Specifically, nut-enriched diets are associated with lowering total cholesterol, LDL-C, and TG compared with control diets. Some RCTs have also shown benefits in reducing ApoB levels and improving the lipoprotein subparticle profile. The major determinant of cholesterol-lowering appears to be nut dose rather than nut type.

As summarized in Figure 1, many bioactive compounds of nuts might explain the beneficial effects of nut consumption on blood lipids and lipoproteins. Improvement in blood lipids is attributable mainly to the favorable fatty acid profile of nuts, but other nut components, namely dietary fiber, phytosterols, and bioactive polyphenols play a role.

Although more research is needed to better understand the biological mechanisms of cardiometabolic protection by nuts, increasing their consumption as part of a healthy diet improves cardiovascular risk factors and helps to reduce the risk of CVD in the general population as well as in individuals at high CVD risk. It goes without saying that an integral step for increasing nut consumption is to effectively educate consumers about the health benefits of nuts and, importantly, communicate how to substitute them for unhealthy foods in the diet to achieve the greatest possible CVD benefits.

## Figures and Tables

**Table 1:** Summary of meta-analyses on the effect of nuts and peanuts on lipid and lipoprotein biomarkers.

Publication	Search Dates	Population	Study Design	Sample Size	Duration of Intervention	Intervention	Control	Outcome Measures	Results Mean Change in mmol/L (95%CI)
Phung, 2009 [13]	through Jul 2008	Non-specified	RCTs with parallel or crossover design	5 RCTs 142 participants	4 weeks	Almonds 25–168 g/d	NCEP step II, usual diet, NCEP step I, high-fat diet, low-fat diet	Lipid profile: TC, LDL-C, HDL-C, TG, LDL/HDL; ApoA-I and apoB Lp(a)	↓ LDL-C −0.18 (−0.34, −0.02) (5 RCTs)
Banel, 2009 [24]	through May 2008	All patient populations and age groups	RCTs with parallel or crossover design	13 RCTs 365 participants	4–24 weeks	Walnuts 15–108 g/d	Controlled diet, Western diet, Med diet, modified low-fat diet, habitual diet, low-fat diet, cholesterol lowering meals	Lipid profile: TC, LDL-C, HDL-C, TG	↓ TC −0.27 (−0.38, −0.15) ↓ LDL-C −0.24 (−0.34, −0.14) (11 RCTs)
Sabaté, 2010 [12]	1992–2004	No recent exposure to lipid-lowering medications	Controlled trials; duration of intervention ≥ 3 weeks; no body weight change between diets at the end of intervention	25 trials 583 participants (pooled analysis with individual participant data)	3–8 weeks	Tree nuts and peanuts 34–100 g/d	Western diet, Med diet, low total, and saturated fat	Lipid profile: TC, LDL-C, HDL-C, TG	↓ TC −0.28 (−0.36, −0.2) ↓ LDL-C −0.26 (−0.34, −0.19) (25 trials)
Mejia, 2014 [16]	through Apr 2014	Non-specified	RCTs; duration of intervention ≥ 3 weeks	47 RCTs 2211 participants	3 weeks–18 months	Tree nuts (almonds, Brazil nuts, cashews, hazelnuts, macadamia nuts, pecans, pine nuts, pistachios, walnuts, and mixed nuts) 30–85.5 g/d	Habitual diet, diet for diabetes, Western diet, low-fat diet, muffin, NCEP step I diet, AHA step 1 diet, ad libitum diet, NCEP step II diet, NCEP step II diet + muffin, cheese	At least one criterion of MetS (waist circumference, TG, HDL-C, blood pressure, glycemic control)	↓ TG −0.06 (−0.09, −0.03) (43 RCTs)
Del Gobbo, 2015 [22]	through Mar 2013	Free of known CVD; Not receiving medication for diabetes, obesity, MetS, hypertension or hyperlipidemia; ≥18 yo	Randomized and nonrandomized controlled trials with parallel or crossover design	42 RCTs and 18 nonrandomized trials 2582 participants	3–26 weeks	Tree nuts 5–100 g/d	Habitual diet, healthy diet, low-fat diet, high-CHO diet, olive oil diet, habitual diet + red meat, low saturated fat diet with cereals and canola oil, AHA step 1 diet, American diet, isocaloric controlled diet, NCEP step 1 or 2 diet, salted pretzels, isocaloric high cholesterol diet, NCEP step 1, Med diet, AHA step 1 diet, ADA diet (with and without nuts)	Lipid profile: TC, LDL-C, HDL-C, TG; apolipoproteins	↓ TC −0.09 (−0.11, −0.07) ↓ LDL-C −0.11 (−0.13, −0.09) (38 RCTs) ↓ ApoB (g/L) −0.042 (−0.065, −0.026) (13 RCTs)
Guasch-Ferré, 2018 [23]	through Jan 2018	Adults	RCTs with a parallel or crossover design; Duration of intervention ≥ 3 weeks	26 RCTs 1059 participants	4 weeks–1 year	Walnuts 15–108 g/d	ad libitum control diet, Med diet, ADA diet, low-fat diet, habitual diet, controlled diet (walnut-free)	At least one of the lipid markers: TC, LDL-C, HDL-C, TG apolipoproteins	↓ TC −0.18 (−0.24, −0.12) ↓ LDL-C −0.14 (−0.2, −0.09) ↓ TG −0.05 (−0.1, −0.01) (23 RCTs)
Lee-Bravatti, 2019 [19]	2015-June 2017 for lipid outcomes	Healthy or with CVD risk factors; ≥18 yo	RCTs; Duration of intervention ≥ 3 weeks	15 RCTs 534 participants	4–16 weeks	Almonds 37–113 g/d	NCEP step II diet, low-fat diet, high-fat diet custom diet, Med diet, NCEP step I diet, ADA diet	Lipid profile: TC, LDL-C, HDL-C, TG, TC/HDL, HDL/LDL; apolipoproteins, Lp(a)	↓ TC −0.28 (−0.43, −0.12) ↓ LDL-C −0.15 (−0.26, −0.05) (13 RCTs)
Morvaridzadeh, 2020 [28]	through June 2019	Non-specified	RCTs with a parallel or crossover design	3 RCTs 384 participants	4–12 weeks	Cashews 28–108 g	Isocaloric diet, baked potato chips	Lipid profile: TC, LDL-C, HDL-C, TG	No change
Liu, 2020 [18]	through June 2019	≥18 yo	RCTs; duration of intervention ≥ 3 weeks	34 RCTs 1677 participants	3–24 weeks	Tree nuts (walnuts, pistachios, hazelnuts, cashews, or almonds) 15–168 g/d	Control diet (nut-free)	Lipid profile: TC, LDL-C, HDL-C, TG	Walnut-enriched ↓ LDL-C −0.09 (−0.12, −0.07) ↓ TG −0.09 (−0.11, −0.07) (16 RCTs) Pistachio-enriched ↓ LDL-C −0.17 (−0.28, −0.06) Hazelnut-enriched No change Almond-enriched No change
Jalali, 2020 [20]	through Nov 2019	≥18 yo	RCTs	3 RCTs 392 participants	4–12 weeks	Cashews 30–42 g/d	Diet for diabetes, isocaloric controlled diet (nut-free)	Lipid profile: TC, LDL-C, HDL-C, TG	No change
Hadi, 2021 [27]	through June 2019	≥18 yo	RCTs; duration of intervention ≥ 3 weeks	12 RCTs 771 participants	3–24 weeks	Pistachios 32–126 g/d	Control diet	Lipid profile: TC, LDL-C, HDL-C, TG	↓ TC −0.19 (−0.33, −0.06) (10 RCTs) ↓ LDL-C −0.1 (−0.14, −0.06) (12 RCTs) ↓ TG −0.13 (−0.16, −0.09) (10 RCTs)
Asbaghi, 2021 [25]	through Sept 2020	Healthy or otherwise; ≥18 yo	RCTs with a parallel or crossover design; Duration of intervention ≥ 3 weeks	27 RCTs 2049 participants	3–77 weeks	Almonds 10–168 g/d	No almond consumption or dietary substitutions containing no almond were used	At least one of the lipid markers: TC, LDL-C, HDL-C, TG	↓ TC −0.13 (−0.2, −0.05) ↓ TG −0.08 (−0.13, −0.02) (27 RCTs) ↓ LDL-C −0.15 (−0.23, −0.07) (26 RCTs)
Wang, 2021 [29]	through Jan 2020	Adults with T2DB	RCTs with a parallel or crossover design; Duration of intervention ≥ 2 weeks	5 RCTs 120 participants	3–12 weeks	Almonds 30–60 g/d	Control diet, NCEP step II diet, peanuts, sunflower kernels	Lipid profile: TC, LDL-C, HDL-C, TG	No change
Xia, 2021 [11]	through June 2021	Patients with T2DB	RCTs	16 RCTs 1041 participants	6–52 weeks	Peanuts and tree nuts (walnuts, pistachios, macadamia nuts, pecans, cashews, almonds, hazelnuts, pine nuts, and Brazil nuts) 6–128 g/d	High-fat diet, low-fat diet, normal-fat diet, habitual diet, diet for diabetes, ADA meal plan (nut-free)	Lipid profile: TC, LDL-C, HDL-C, TG	↓ TC −0.14 (−0.26, −0.02) (14 RCTs) ↓ TG −0.1 (−0.17, −0.02) (12 RCTs)
Moosavian, 2022 [15]	through Mar 2021	Patients with T2DB; ≥18 yo	RCTs; Duration of intervention ≥ 3 weeks	9 RCTs 264 participants	4–12 weeks	Almonds 29–113g/d	NCEP step II diet, cheese, raw peanut with low carbohydrate diet, high-fat diet, low-fat diet, sunflower kernels with diabetic diet, custom diet (almond-free)	Lipid profile: TC, LDL-C, HDL-C, TG	↓ LDL −0.14 (−0.26, −0.02) (8 RCTs)
Arabi, 2022 [26]	through Dec 2021	Diagnosed with MetS; ≥18 yo	RCTs with a parallel or crossover design	8 RCTs 506 participants	4–112 days	Walnuts (all forms, plain, or walnut-fortified food) 30 g–108 g/d	Standardized shakes, control diet, isocaloric white bread, ad libitum diet without walnuts, lifestyle counseling	Lipid profile: TC, LDL-C, HDL-C, TG	↓ TG −0.17 (−0.32, −0.03) (5 RCTs)
Mates, 2022 [17]	through Nov 2021	Middle-aged and older adults ≥40 yo or mean age ≥50 yo	RCTs with a parallel or crossover design; Duration of intervention ≥ 3 weeks	17 RCTs 2466 participants	4 weeks-2 years	Walnuts (including plain or walnut-fortified food) 19.3–75 g/d	Med diet, modified low-fat diet, Western-type diet, habitual diet, CKD patients’ diet (walnut-free)	Lipid profile: TC, LDL-C, HDL-C, TG	↓ TC −0.13 (−0.2, −0.07) ↓ LDL-C −0.15 (−0.2, −0.11) (12 RCTs) ↓ TG −0.08 (−0.12, −0.04) (13 RCTs)
Eslami, 2022 [21]	through Apr 2021	Overweight/obese (BMI: 25–40 kg/m^2^); free of chronic diseases; ≥18 yo	RCTs with a parallel or crossover design; Duration of intervention ≥ one week	10 RCTS 711 participants	4–72 weeks	Peanuts and tree nuts (almonds, walnuts, hazelnuts, pistachios, cashews, macadamia nuts, Brazil nuts, pine nuts, pecans, mixed nuts) 20–60 g/d	Isocaloric nut-free diet	At least one of the following: Serum lipid profile: TC, LDL-C, HDL-C, TG	↓ TG −0.15 (−0.29, −0.01) (9 RCTs)
Parilli-Moser, 2022 [14]	through July 2021	Healthy or with MetS or at high risk of MetS	RCTs	9 RCTs 643 participants	2–24 weeks	Peanuts, peanut butter or high oleic acid peanuts 25–200 g/d	Hypocaloric diet, habitual diet, ADA meal plan, substitute snack (grain bar, white rice bar, candy, or almonds) (peanut-free)	Lipid profile: TC, LDL-C, HDL-C, TG	↓ TG −0.13 (−0.2, −0.07) (9 RCTs)

RCT, randomized controlled trial; NCEP, National Cholesterol Education Program; TC, total cholesterol; LDL-C, low-density lipoprotein cholesterol; HDL-C, high-density lipoprotein cholesterol; TG, triglycerides; CHO, carbohydrates; ANDEAP, Academy of Nutrition and Dietetics Evidence Analysis Process; AHA, American Heart Association; ADA, American Diabetes Association; Med Diet, Mediterranean diet; BW, body weight; WC, waist circumference; BMI, body mass index; Lp (a), lipoprotein (a); MetS, metabolic syndrome; T2DB, type 2 diabetes. Units for blood lipids are presented as mmol/L, except for ApoB which is presented as g/L. Only lipid outcomes are reported.

## Data Availability

No new data were created or analyzed in this study. Data sharing is not applicable to this article.

## Abbreviations

ALA: α-linolenic acid; Apo, apolipoproteins; BMI, body mass index; CHD, coronary heart disease; CVD, cardiovascular diseases; FFQ, food frequency questionnaire; HDL, high-density lipoprotein cholesterol; HMG-CoA, 3-hydroxy-3-methylglutaryl-CoA; HNF-4α, hepatocyte nuclear factor; LDL, low-density lipoprotein cholesterol; Lp(a), Lipoprotein(a); LXR, liver-X-receptor; MD, mean difference; MUFA, monounsaturated fat; NFκB, nuclear factor kappa B; PPARγ, peroxisomal proliferator-activated nuclear receptors gamma; PREDIMED, PREvención con DIeta MEDiterránea; PUFA, polyunsaturated fat; RCT, randomized controlled trial; SFA, saturated fat; SRBPs, sterol-regulatory element binding proteins; TG, triglycerides; TC, total cholesterol; VLDL, very low-density lipoprotein; WAHA, Walnuts and Healthy Aging Study.
